# Multilevel Analysis of the Nutritional and Health Status among Children and Adolescents in Eastern China

**DOI:** 10.3390/nu14040758

**Published:** 2022-02-11

**Authors:** Ting Tian, Yuanyuan Wang, Wei Xie, Jingxian Zhang, Yunlong Ni, Xianzhen Peng, Guiju Sun, Yue Dai, Yonglin Zhou

**Affiliations:** 1 Institute of Food Safety and Assessment, Jiangsu Provincial Center for Disease Control and Prevention, Nanjing 210009, China; jstt@jscdc.cn (T.T.); wyypro@foxmail.com (Y.W.); jscdcxiewei@sina.com (W.X.); z18252063009@163.com (J.Z.); nyl2008@163.com (Y.N.); 18915999341@163.com (Y.D.); 2 Department of Nutrition and Food Hygiene, School of Public Health, Southeast University, Nanjing 210009, China; gjsun@seu.edu.cn; 3 Department of Public Health and Preventive Medicine, Kangda College of Nanjing Medical University, Lianyungang 222000, China; xianzhenpeng@njmu.edu.cn

**Keywords:** nutritional status, children and adolescents, malnutrition, overnutrition

## Abstract

We aimed to identify multiple nutritional health problems and the relevant factors among children and adolescents aged 7–17 years. This study was part of the China Nutrition and Health Surveillance of Children and Lactating Mothers in 2016–2017, conducted in Jiangsu Province in eastern China. After sampling, 3025 school-age children and adolescents were enrolled into this study. Demographic information collections and anthropometric measurements were conducted by trained local Center for Disease Control and Prevention (CDC) staff. Venous blood in the amount of 6 mL was drawn from each participant in the morning and used for testing biochemical and nutritional indicators. Multivariate logistic regression analysis and Poisson regression analysis were used for overnutrition- and undernutrition-related disorders to test relevant personal, parental, and household factors. The prevalence of wasting, overweight, and obesity was 5.5%, 14.8%, and 12.7%, respectively. Metabolic syndrome (MetS) was prevalent among 5.1% of participants. Among the study participants, 29.5% had hyperuricemia. The overall prevalence of high low-density lipoprotein (LDL) and high total cholesterol (TC) of all participants was 4.8% and 7.4%, respectively. 0.9% of the participants had vitamin A deficiency (VAD) and 14.6% had marginal vitamin A deficiency; 25.1% had vitamin D deficiency (VDD) and 54.5% had inadequate vitamin D levels. Anemia was present in 4.0% of all participants. The prevalence of zinc deficiency was 4.8%. Demographic characteristics, behavioral characteristics, parents’ characteristics, and family characteristics were associated with these multiple malnutrition disorders. The double burdens of malnutrition, which includes overnutrition- and undernutrition-related diseases, were prevalent among the school-age children and adolescents in Jiangsu Province in eastern China. There were various factors related to different nutritional problems. Thus, health education focusing on behavior intervention and nutrition education are necessary in containing nutritional problems among children.

## 1. Introduction

Nutrition is essential for children’s neurodevelopment and reproductive development and is associated with linear growth [1]. Improving children’s nutrition is urgently needed for global development and for reducing the burden of noncommunicable diseases (NCDs) [2]. Nutritional conditions in the early stages of life may have both short-term and longer lasting effects, including an increased risk of NCDs and other chronic diseases [3]. Cardiovascular diseases, which are the most common causes of mortality among adults worldwide, are rooted in childhood, underlying the urgency of identifying and intervening with at-risk children [4,5].

Further, approximately one-half of all childhood deaths globally are caused directly or indirectly by malnutrition [6]. Various malnutrition diseases, including undernutrition- and overnutrition-related disorders, are highly prevalent among children and adolescents in many countries [7,8]. For example, vitamin A deficiency (VAD) remains a public health problem in China, where the prevalence of VAD in children was 5.16% in 2015 [9]. A high prevalence of vitamin D deficiency (VDD) has also been reported in infants, children, and adolescents from diverse countries around the world [10]. The overall prevalence of anemia was 8.8% among children in Hunan Province in China [11] and 17.49% among school-age children in Latin America and the Caribbean [12]. Zinc is an essential trace element for growth and development in children, but zinc deficiency is a serious nutritional problem in China. In 2012, the China Nutrition and Health Survey showed a 6.8% prevalence of zinc deficiency in 3407 school-age children [13].

In addition to undernutrition, overnutrition is a serious problem. The results of five consecutive national surveys from 1995 to 2004, as part of the Chinese National Survey on Students’ Constitution and Health, showed that the main growth problems had changed from undernutrition to overnutrition in the past 20 years [14]. The nutrition issues of children and adolescents have become more important in view of the global epidemic of obesity, metabolic syndrome (MetS), and other metabolic risk factors. Worldwide, more than 100 million children are obese [15].

One predictor of future health risk is Metabolic syndrome (MetS), a cluster of cardiovascular risk factors that include abdominal obesity, which is mainly measured by high waist circumference (WC), hypertension, high fasting triglycerides (TG), low high-density lipoprotein (HDL) cholesterol, and high fasting blood glucose (FBG) [16]. The recent prevalence of MetS in American children and adolescents was 9.83%, according to the results of National Health and Nutrition Examination Survey (NHANES) [17]. Data from the China Health and Nutrition Surveys (CHNS) showed that the prevalence of MetS in Chinese children and adolescents aged 7–18 years was 3.37% [18]. Meanwhile, recent evidence suggests that hyperuricemia is at a high level in children and adolescents in both China and America, with the prevalence ranging from 10.1% to 26.02% [19,20,21].

With sustained economic development, the living standards of Chinese residents, and especially residents of Jiangsu Province, which is a relatively developed area in the country, have continuously improved. However, China still faces many nutritional problems, such as the coexistence of undernutrition- and overnutrition-related disorders among children and adolescents and the lack of clarity regarding the sociodemographic factors that are related to these nutritional problems. Sociodemographic differences play important roles in nutrition status, especially among children and adolescents [22,23,24]. To date, there have been few studies conducted in Jiangsu Province to describe comprehensively the nutritional status of school-age children and adolescents. Thus, this study aimed to identify the multiple nutritional health problems among children and adolescents aged 7–17 years in Jiangsu Province in China, and the relevant factors contributing to those problems, based on provincial representative data.

## 2. Materials and Methods

### 2.1. Study Participants

This study was part of the China Nutrition and Health Surveillance of Children and Lactating Mothers in 2016–2017. We selected school-age children and adolescents from 12 survey sites, including urban and rural areas in Jiangsu Province in eastern China. The representative provincial samples for accessing the nutritional and health status of school-aged participants were randomly selected by multistage stratified cluster random sampling methods. The study design and sampling methods were described by Dongmei Yu et al. in an article introducing the China Nutrition and Health Survey from 1982–2017 [25]. 

In the first stage of the study, 12 survey sites were randomly picked from the whole province, including two survey sites in large cities, eight sites in medium cities, and two sites in generally rural counties. In the second stage, two townships/subdistricts were randomly picked from each survey site. In the third stage, two communities or villages of township/subdistricts were randomly selected. In the last stage, 70 school-age children and adolescents were randomly picked from the selected communities or villages. There were at least 280 children aged 7–17 years, with equal numbers of males and females, selected in each of the survey sites. Replacements were allowed only from the same village/neighborhood and similar households as those of the original participants. The replacement rate had to be less than 35%. After sampling, 3360 school-aged children and adolescents were enrolled into the study. In the final analysis, 3025 participants were included, after excluding participants who were unable to provide key information, such as sociodemographic characteristics, lab-testing information, or anthropometric results (n = 335).

### 2.2. Ethics Approval and Consent to Participate

This study was performed according to the Declaration of Helsinki and approved by the ethical committee of the China Center for Disease Control and Prevention (the China CDC); the corresponding ethical approval number was 201614. All of the participants agreed to take part in this study and signed the required informed consent form.

### 2.3. Data Collection and Measurements

Interviews were based on household and individually structured questionaries provided by the China CDC project group, to collect demographic information and additional information concerning physical activities, screen time, parents, household sizes, and other details. Interviews were conducted by a strictly trained investigator in a face-to-face manner. 

Screen time refers to the time using an electronic screen every day. A low level of screen time was defined as <2 h and a high level was defined as ≥2 h. The assessment of physical activities was based on reaching a level of coarsened breathing or a heart rate rise for more than 30 min. An exercise level of 0–3 days per week was defined as a low physical activity level; an exercise level for more than 4 days per week was defined as a high physical activity level.

Anthropometric measurements including height, weight, WC, and blood pressure were conducted by trained local CDC staff. All equipment used in the measurements were selected according to the guidance of the national project group. Height and weight were measured without shoes or coats. A stadiometer (TZG, Nantong, China) was used for height measurement, with an accuracy to 0.1 cm. The equipment used for weight measurement was an electric scale (TANITA HD-390, Tokyo, Japan) and the results were accurate to 0.1 kg. A soft tape was used to measure WC and the participants were measured in fasting states, at the midpoint between the bottom of the rib cage and the uppermost border of the iliac crest, following exhalation. An electronic sphygmomanometer (Omron HBP 1300, Kyoto, Japan) was used for the measurement of blood pressure. Each participant was measured three times for blood pressure, in 5 min intervals, and the average value was recorded.

Laboratory sample collection and tests included the collection of participants’ fingertip blood for testing hemoglobin by a Hemocue 201^+^ Hemoglobin Analyzer (HemoCue, Angelholm, Sweden). In addition, 6 mL of fasting venous blood was drawn from participants in the morning and used for testing biochemical and nutritional indicators. Blood glucose, lipids, uric acid, serum vitamin A, vitamin D, and zinc, etc., were detected according to the standard methods provided by the China CDC project group.

### 2.4. Diagnostic Criteria and Definitions

The Chinese “screening standard for malnutrition of school-age children and adolescents” was applied to screen wasting among children by age-specific values [26].

The Chinese “screening for overweight and obesity among school-age children and adolescents” was used to identify overweight and obesity by age-specific values [27]. 

MetS was diagnosed according to the modified criteria of the National Cholesterol Education Program-Adult Treatment Panel III (NCEP-ATP III) for children and adolescents aged 7–17 years [28]. When participants had more than three of the following five components, they were diagnosed as MetS:

(1) Abdominal obesity, where WC ≥ age- and gender-specific 90th percentile [29];
(2) Elevated triglyceride (TG), where TG ≥ 1.24 mmol/L;
(3) Low high-density lipoprotein (HDL), where HDL ≤ 1.03 mmol/L;
(4) Elevated blood pressure, where systolic blood pressure (SBP) or diastolic blood pressure (DBP) ≥ 90th percentile for gender, age, and height [30];
(5) Elevated fasting blood glucose (FBG), where glucose ≥ 6.1 mmol/L.

The following diagnostic criteria and definitions were applied in this study:

Elevated total cholesterol (TC): TC ≥ 5.18 mmol/L [31]; elevated low high-density lipoprotein (LDL): LDL ≥ 3.36 mmol/L [31];

Hyperuricemia: serum uric acid (SUA) ≥ 357 μmol/L [21];

Vitamin A deficiency (VAD), marginal deficiency in vitamin A (VAMD), and vitamin A sufficiency were referred to serum retinol levels less than 0.2 μg/mL, 0.2–0.3 μg/mL, and more than 0.3 μg/mL, respectively [32];

Vitamin D deficiency (VDD), inadequacy of vitamin D (VDI), and vitamin D sufficiency were defined by serum 25(OH)D levels less than 12 ng/mL, 12–20 ng/mL, and more than 20 ng/mL, respectively [33]; 

Anemia: for participants aged 5–11 years, hemoglobin < 115 g/L; for participants aged 12–14 years, hemoglobin < 120 g/L; for male participants aged 15–17 years, hemoglobin < 130 g/L; for female participants aged 15–17 years, hemoglobin < 120 g/L [34];

Zinc deficiency in children and adolescents was defined as serum zinc concentrations < 76.5 μg/dL [35].

The number of overnutrition-related disorders included a count of five MetS components (abdominal obesity, high TG, low HDL, elevated blood pressure, and elevated FBG) and hyperuricemia, high LDL, high TC. The number of undernutrition-related disorders included a count of prevalent vitamin A insufficiency, vitamin D deficiency, anemia, and zinc deficiency.

### 2.5. Statistical Analysis

All statistical analysis was conducted on R software (Version 4.1.0, developed by R Development Core Team, originated from the Statistics Department of the University of Auckland, Auckland, New Zealand). According to the normality of the continuous variables, they were displayed as either median (interquartile range, IQR) or average (standard deviation, SD). Pearson’s chi-square test was used to compare the distribution of different characteristics and metabolic risk factors between gender groups. Multivariate logistic regression analysis was applied to find the related factors for multiple nutritional problems in the research populations. 

In order to understand the relevant factors for children’s and adolescents’ nutritional status, we conducted the multivariate logistic regression analysis by including the following potential factors in the analysis: participants’ demographic characteristics, behavioral characteristics, parents’ characteristics, and family characteristics. Multivariate Poisson regression analysis was carried out for the overnutrition- and undernutrition-related disorders to test the participants’ personal, parental, and household factors. A two-sided *p* < 0.05 was utilized to define statistical significance.

## 3. Results

### 3.1. Characteristics of Research Populations

Of the 3025 school-age children and adolescents (7–17 years old) in the study, there were 50.2% males and 49.8% females; 34.6% of the participants were from urban areas and 65.4% were from rural areas. As displayed in Table 1, the distribution of most characteristics, such as age, age group, living area, physical activity level, screen time, age of mother, age of father, education of mother, and education of father, were not significantly different between male and female participants (all *p* > 0.05). There was a remarkable difference in household size between genders (*p* = 0.009). When it came to anthropometric indexes, male participants had higher height, weight, and SBP than female participants (all *p* < 0.05), except for DBP, which was not different between males and females (Table 2). There were noticeable differences in many biochemical indices. Male participants had higher levels of FBG, serum uric acid, vitamin D, and serum zinc than did females (all *p* < 0.05). The TG, TC, LDL, and HDL of female participants were at higher levels than they were males (all *p* < 0.05). However, there was no significant difference in vitamin A level between genders (*p* = 0.963), as shown in Table 2. 

As noted above, screen time refers to the time of using an electronic screen every day. A low level of screen time is defined as <2 h and a high level of screen time is defined as ≥2 h. The assessment of physical activities was based on reaching a level of coarsened breathing or a heart rate rise for more than 30 min. An exercise level of 0–3 days per week was defined as a low physical activity level; an exercise level of more than 4 days per week was defined as a high physical activity level. 

### 3.2. Nutritional and Health Statuses of School-Age Children and Adolescents

**Figure 1:**
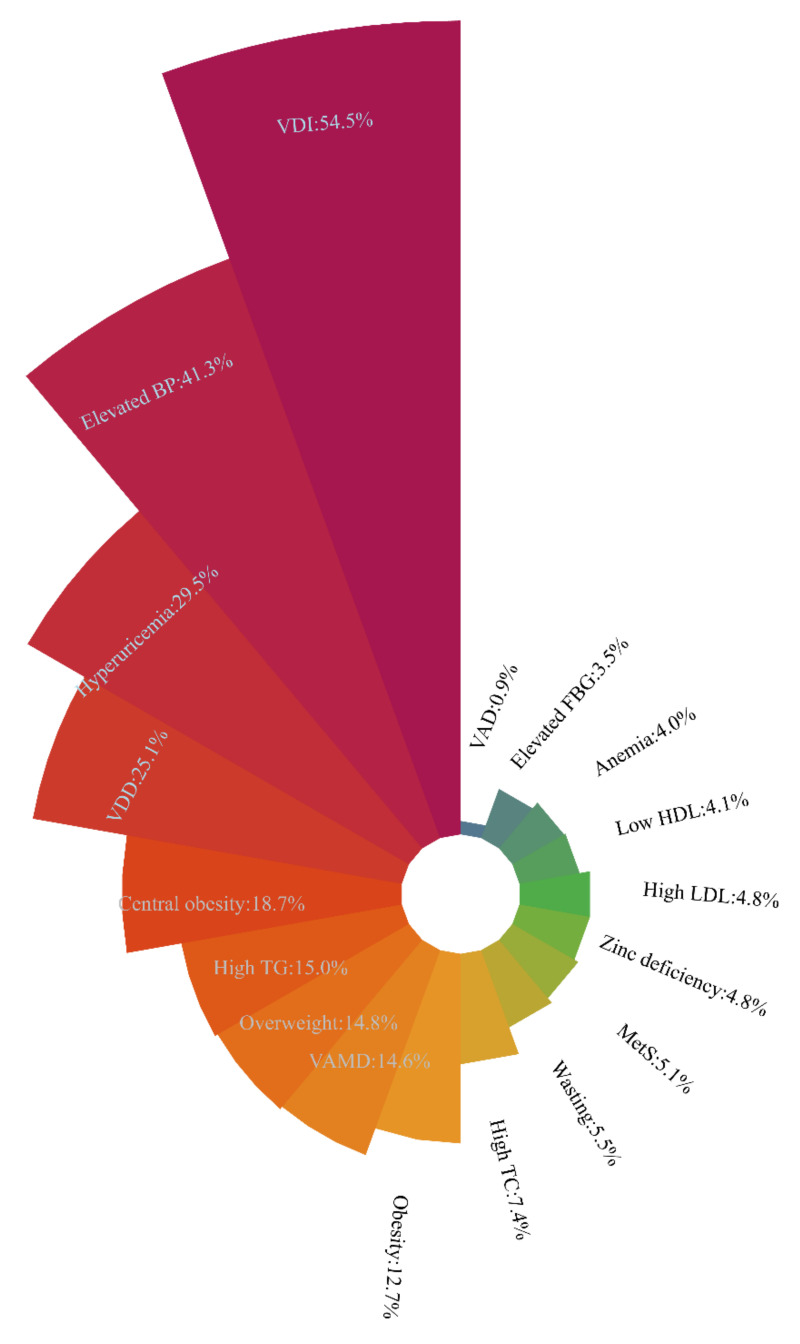
Sequence of prevalence of the relevant nutritional and health problems among school-age children and adolescents. VDI = vitamin D inadequacy; elevated BP = elevated blood pressure; VDD = vitamin D deficiency; high TG = high triglyceride; VAMD = vitamin A marginal deficiency; high TC = high total cholesterol; MetS = metabolic syndrome; high LDL = high low-density lipoprotein; low HDL = low high-density lipoprotein; elevated FBG = elevated fasting blood glucose; VAD = vitamin A deficiency. MetS and its five components (central obesity, high TG, low HDL, elevated BP, and elevated FBG) were diagnosed according to the modified criteria of the National Cholesterol Education Program-Adult Treatment Panel III (NCEP-ATP III) for children and adolescents aged 7–17 years.

The nutritional and health statuses of school-age children and adolescents are displayed in Figure 1 and Table 3. Figure 1 is presented according to the sequences of the prevalence of the relevant nutritional and health problems. The prevalence of wasting, overweight, and obesity were 5.5%, 14.8%, and 12.7%, respectively. MetS was prevalent among 5.1% of the participants. The prevalence of the five MetS components, including abdominal obesity, high TG, low HDL, elevated blood pressure, and elevated FBG, were 18.7%, 15.0%, 4.1%, 41.3%, and 3.5%, respectively. Among the study participants, 29.5% had hyperuricemia. The prevalence of high LDL among all participants was 4.8%. The prevalence of high TC among all participants was 7.4%; 0.9% of the participants were VAD, 14.6% had marginal vitamin D deficiency, and 84.5% were sufficient in vitamin A levels; 25.1% of the participants had VDD, 54.5% had inadequate VDD, and 20.4% were sufficient in vitamin D levels. The prevalence of anemia among all participants was 4.0%. The prevalence of zinc deficiency was 4.8%. There were significant differences between genders in the prevalence of weight groups, MetS, hyperuricemia, high TC, and vitamin D conditions, as shown in Table 3.

After age group stratification, we compared the nutritional status within each age group and between the age groups, as shown in the Supplementary Materials, Table S1. The proportions for weight groups, vitamin A, and vitamin D were significantly different between participants in the 7–12 years age group and the 13–17 years age group (*p* < 0.001). The 13–17 years age group had a significant higher possibility of hyperuricemia and anemia than did the 7–12 years age group (both *p* < 0.05).

### 3.3. Related Factors Associated with Various Nutritional Diseases

In Table 4, the multivariate logistic regression of multiple nutritional diseases (MetS, hyperuricemia, vitamin A insufficiency, vitamin D deficiency, anemia, and zinc deficiency) are displayed. Table 4 includes the most common factors of demographic characteristics, behavioral characteristics, parents’ characteristics, and family characteristics. These common factors were considered in the multivariate logistic analysis. The adjusted odds ratios (AORs), along with their 95% confidence intervals (95% CI) and *p* values, are portrayed in Table 4.

We found that age groups and weight groups were significantly associated with MetS. Compared to the 7–12 years age group, the 13–17 years age group was more prone to have MetS (AOR = 1.79, 95% CI: 1.17–2.73, *p* = 0.007). Participants in the overweight groups were more likely to have MetS (OR = 7.47, 95% CI: 4.26–13.11, *p* < 0.001), compared to those in the reference group (in terms of wasting and normal weight). Furthermore, participants in the obesity group were more likely to have MetS than were those in the reference group (OR = 41.14, 95% CI: 25.05–67.56, *p* < 0.001). 

With respect to hyperuricemia, a participant’s sex, age group, weight group, age of the mother, and education of the father were relevant factors. Females had significantly less likelihood of hyperuricemia than did males (OR = 0.30, 95% CI: 0.25–0.36, *p* < 0.001). Participants in the 13–17 years age group had a higher possibility of hyperuricemia than did those in the 7–12 years age group (OR = 5.09, 95% CI: 4.13–6.27, *p* < 0.001). The overweight and obesity groups were both higher in the prevalence of hyperuricemia than were other groups (OR = 1.94, 95% CI: 1.53–2.46, and OR = 4.38, 95% CI: 3.38–5.67, respectively). Compared to the participants whose mothers were younger than 37 years, the participants whose mothers were older than 37 years were more likely to have hyperuricemia (OR = 1.46, 95% CI: 1.13–1.89, *p* = 0.004). Participants whose father’s education was university and above were less likely to have hyperuricemia than were those whose father’s education was primary school and below (OR = 0.63, 95% CI: 0.41–0.97, *p* = 0.035).

When focusing on vitamin A insufficiency, a participant’s age group, weight group, screen time, and education of the mother were the relevant factors. Participants in the 13–17 years age group were less likely to have a prevalence of vitamin A insufficiency, when compared with the those in the 7–12 years age group (OR = 0.34, 95% CI: 0.26–0.45, *p* < 0.001). Participants who were overweight had less risk of vitamin A insufficiency than did those in the reference group (in terms of wasting and normal weight, OR = 0.63, 95% CI: 0.46–0.87). Obesity subjects also had less risk of vitamin A insufficiency than did those in the reference group (OR = 0.37, 95% CI:0.25–0.55, *p* = 0.005). In addition, participants with higher screen time were prone to have vitamin A insufficiency (OR = 1.45, 95% CI: 1.09–1.94, *p* = 0.011). Children and adolescents whose mothers had higher education levels were less likely to have vitamin A insufficiency, compared to those whose mothers had primary school education or below; the OR were 0.64 and 0.55, respectively. 

With reference to VDD, a participant’s sex, age group, physical activity, screen time, education of the mother, and household size were the relevant factors. Females were more likely to have VDD than males, with AOR = 1.80, 95% CI: 1.51–2.15, *p* < 0.001. The 13–17 years participants had a higher risk of being VDD than did the 7–12 years participants (OR = 2.53, 95% CI:2.09–3.08, *p* < 0.001). Remarkably, participants with a high level of physical activity were less likely to have VDD (AOR = 0.69, 95% CI: 0.58–0.83, *p* < 0.001). Subjects with high screen time were associated with an increased risk of VDD (AOR = 1.40, 95% CI: 1.09–1.80, *p* = 0.008). In addition, VDD was related to participants’ mothers’ education levels (middle school vs. primary school and below: AOR = 0.68, 95% CI: 0.52–0.89; university and above vs. primary school and below: AOR = 0.58, 95% CI: 0.40–0.86). Those participants whose household size was ≥5 members were more prone to have VDD than were those whose household size was ≤4 members (AOR = 1.25, 95% CI = 1.05–1.49, *p* = 0.014). 

When considering anemia, the sex, age group, and obesity of participants were the relevant factors. Sex was associated with anemia (females were more likely to have anemia, with AOR = 2.95, 95% CI = 1.95–4.51, *p* < 0.001), as well the age group (13–17 years: AOR = 1.82, 95% CI: 1.20–2.77, *p* = 0.005). Inversely, obesity groups were less likely to show prevalence in anemia (AOR = 0.37, 95% CI: 0.15–0.93, *p* = 0.034). For zinc deficiency, participants living in the rural area had a higher risk than did those who lived in urban areas (AOR = 1.80, 95% CI: 1.21–2.68, *p* = 0.004).

### 3.4. The Relevant Factors of Overnutrition- and Undernutrtion-Related Disorders

The Poisson regression analysis took the amount of overnutrition-related disorders (i.e., the prevalent five MetS components and hyperuricemia, high LDL, and high TC) and undernutrition-related disorders (i.e., vitamin A insufficiency, VDD, anemia, and zinc deficiency) as dependent variables to test the personal, parental, and household factors. We found that the factors of sex, age group, and weight group were related to overnutrition-related disorders, seen in Table 5. Females were less likely inclined to overnutrition-related disorders (AOR = 0.81, 95% CI: 0.51–0.85, *p* < 0.05). The older age group (13–17 years) and the overweight and obesity participants were more prone to have a variety of overnutrition-related disorders (AOR = 1.44, 1.66 and 2.77, respectively, all *p* < 0.05). With respect to undernutrition-related disorders, we found that a participant’s sex, age, living area, weight group, physical activity, screen time, education of mother, and household size were the relevant factors. Females, older age group (13–17 years), rural areas, higher screen time, and large household size (≥5 members) were significantly and positively associated with the occurrence of undernutrition-related disorders (all AOR > 1). Conversely, participants who were overweight or obese, or who had a high level of physical activity or a mother with higher education, were less prone to clustering in undernutrition-related disorders. (All AOR < 1 and all *p* < 0.05).

## 4. Discussion

The present study illustrated the multiple nutritional statuses of school-age children and adolescents in Jiangsu Province, China. The presence of the double burden of malnutrition was common among the children and adolescents. To be more specific, overnutrition-related diseases such as overweight, obesity, MetS, hyperuricemia, high LDL and high TC, and undernutrition-related diseases (vitamin A insufficiency, VDD, anemia, and zinc deficiency) were prevalent in the 7–17 years age group of participants. Demographic characteristics, behavior characteristics, parents’ characteristics, and family characteristics were associated with different malnutrition disorders.

In this study, 5.5% of school-age children and adolescents in Jiangsu Province showed prevalence in wasting, according to the Chinese standard, and males were more prone to wasting than were females (6.3% vs. 4.7%). Based on data from 2014 Chinese National Surveys on Students Constitution and Health, wasting was present in 5.5% of Chinese children and adolescents, which was consistent with the findings in our study [36]. In present study, the prevalence of overweight and obesity was 14.8% and 12.7%, respectively. The prevalence of both overweight and obesity was higher in males than in females. Results from the 2015 China Health and Nutrition Survey (CHNS) showed that, generally, overweight prevalence was 15.43% and obesity prevalence was 11.06% among children and adolescents aged 6 to 17 years [37]. Data from NHANES indicated that obesity prevalence among America youth aged 2–19 years, from 2015–2016, was 18.5% [38]. 

Overweight and obesity have become a serious nutritional problem in children all around the world [39,40]. The prevalence of MetS in this study’s participants was 5.1%, while the results of the CHNS yielded an overall 3.37% prevalence of MetS [18]. The Jiangsu Province’s level of prevalence of MetS in school-age children and adolescents was higher than the national average. This indicated that urgent measures must be taken to contain MetS. Among school-age children and adolescents, 29.5% had hyperuricemia (≥357 μmol/L). The overall prevalence of hyperuricemia among American adults had increased from 18.0% in 1988 to 21.4% in 2008 [41], which suggested a high prevalence level of hyperuricemia in a large number of children and adolescents.

In our study, we found VAD and marginal deficiency in vitamin A were prevalent, respectively, in 0.9% and 14.6% of the participants. Similarly, the national results of the China Nutrition and Health Surveillance of Children and Lactating Mothers, in 2016–2017, showed that the prevalence of VAD and the marginal deficiency of vitamin A was 0.96% and 14.71%, respectively [42]. The prevalence of VAD and marginal deficiency in vitamin A among school-age children and adolescents in Jiangsu Province were at the national average level. Based on these findings, vitamin A marginal deficiency was the main form of vitamin A insufficiency in Chinese children. The prevalence of VDD and inadequacy of vitamin D were 25.1% and 54.5%, respectively. In spite of different diagnostic criteria, VDD was very common among school-age children [43,44]. Sufficient sunlight, dietary vitamin D and vitamin D supplements are recommended for children.

Our study indicated that the presence of anemia in children and adolescents aged 7–17 years was 4.0%. Anemia was more common in girls than in boys, at 6.0% vs. 2.0%, respectively. These results were consistent with data from the Chinese National Nutrition and Health Survey (CNNHS) [45], where 4.8% of children had zinc deficiency and the prevalence of zinc deficiency was not different between genders. Zinc is an essential microelement for growth and development in children, but zinc deficiency is still a serious nutritional problem, especially in school-age children [13]. These findings indicated that multiple overnutrition-related diseases and undernutrition-related diseases coexisted in the surveyed areas.

In addition, we analyzed the roles of different factors, such as demographic characteristics, behavioral characteristics, parents’ characteristics, and family characteristics in the development of different malnutrition diseases. Age and weight are related to MetS. Participating adolescents over 13 years, obese participants, and overweight participants are more likely to suffer from MetS. Other research has also shown that age and obesity can increase the risk of developing MetS [46]. These results indicate that more attention should be paid to obese and overweight children, and to adolescents aged 13–17 years.

Based on our study, age, weight and the age of mothers were positively associated with hyperuricemia. Females and children whose mothers had high education levels were less likely to have hyperuricemia. The level of uric acid increased with age in children aged 5–14 years in southeast China [47]. One study showed that boys had higher mean serum uric acid concentrations than girls [19], and this difference was consistent with our results. Age and education of the mother were new influencing factors for hyperuricemia in children. These results support the view that family factors, especially factors pertaining to mothers, may have some influence on the development of hyperuricemia in children.

When focusing on vitamin A insufficiency, age, weight, screen time, and the education of mothers are relevant factors. Older, overweight, and obese children, and those whose mothers have higher education levels, are less likely to be insufficient in vitamin A. Similarly, a meta-analysis reported that a prevalence of VAD and marginal VAD both decreased with increasing age in Chinese children [9]. Furthermore, a Chinese nationally representative study reported that the average of serum retinol concentration was notably higher in over-nutrified than non-over nutrified children and adolescents [48].

The screen time of children was positively associated with vitamin A insufficiency. This can be understood by the fact that vitamin A is the key nutrient implicated in maintaining the function of normal vision; prolonged eye use may consume vitamin A [49,50]. Vitamin D, with its influence on calcium and phosphorus homeostasis, as well as bone health and immunity health, is the essential micronutrient in the growth and development of children [51]. VDD is associated with numerous adverse health issues, such as MetS [52], dyslipidemia [53], and cardiovascular diseases [54]. In our study, sex, age, physical activity, screen time, the education of mothers, and household sizes were related to VDD. The factors of female gender, age more than 13 years, high screen time, and large household size were positively associated with VDD in this population.

One Chinese study investigated VDD among 460,537 children and concluded that the factor of female gender contributed to VDD and vitamin D insufficiency [55]. Girls and boys have different fat distributions and girls have a higher physiological level of estrogens that play certain roles in vitamin D metabolism [56]. Older children have a higher risk of VDD, which can be demonstrated by the facts that muscle mass increases after puberty and muscle is a storage site for vitamin D metabolites [57,58]. Higher screen time means less sun exposure, which can affect vitamin D synthesis [59]. Meanwhile, family factors, such as education of mothers and household sizes, may have some roles in the development of VDD.

In our research, the anemia-related factors were sex, age, and weight. Our results accorded with the CNNHS in 2010–2012, which analyzed the factors associated with anemia in Chinese children and adolescents [45]. That study found that girls and older children were less likely to have anemia and that obese children had a significantly lower prevalence of anemia than did those who had normal or low body weight, which could be due to a better dietary nutritional intake in overweight or obese people [45].

The present study found that children living in rural areas were more likely to be zinc deficient than were those who lived in urban areas. A systematic review of the literature came to the same conclusion [60]. Zinc deficiency may result from low zinc dietary intake and/or the reduction in bioavailability of zinc. Consumption of foods with high phytate content (e.g., rice and wheat) in the rural areas may lead to low zinc absorption and reduced bioavailability [61].

Moreover, the Poisson regression analysis results showed that girls seemed to have a higher risk of undernutrition-related disorders and less risk of overnutrition-related disorders than boys, which was consistent with previous evidence [62]. There is evidence that girls are more vulnerable than boys to disadvantage in nutrient intake, due to China’s “son preference” norm [63]. Novel findings in our study revealed that the ages of children, and adolescents aged 13–17 years, were related to multiple overnutrition and undernutrition disorders. In this study, adolescents aged 13–17 years had a higher prevalence of overweight, MetS, and hyperuricemia. On the other side, adolescents aged 13–17 years had a higher prevalence of VDD and anemia. Therefore, we should pay special attention to the various nutritional and health problems of adolescents in puberty and late puberty. Children’s caregivers in poor areas of China have a lack of feeding information and cannot give their children complementary foods in a proper way, including proper starting times, frequency, and quality [64]. Lower education among rural caregivers was another risk factor in children’s stunting. This result was consistent with previous studies [65].

Furthermore, childhood obesity is linked to serious complications in adulthood, which include an increased risk of ill health and premature death [66]. The high prevalence and dramatically rising trend in childhood obesity suggest that aggressive approaches in prevention and treatment should be pursued to reduce the attendant health and social consequences of obesity. One previous study indicated that children and adolescents aged 6–17 who participate in moderate to vigorous physical activities are likely to experience multiple positive health outcomes [67]. Thus, providing sufficient physical activity time is essential for children’s and adolescents’ health. Screen time, including the daily use of mobile phones, TV watching, and playing computer and video games, was related to the nutritional status of children [68], and especially to vitamin A insufficiency and VDD. For children’s nutrition and vision health, it is necessary to strictly limit everyday screen time. In addition, domestic factors have influences on children’s nutritional health. For example, the significant positive effects of a family economy on the weight of children and adolescents have been gradually and persistently increasing [69].

It is noted that this study does have some limitations. First, this study did not take into consideration dietary factors and some other influences, such as environmental factors or genetic susceptibility; however, these factors may raise issues affecting different nutritional and health problems among children and adolescents. In future studies, we will further explore the roles of these factors in the nutritional status of children. Second, this study, as a cross-sectional design, only provides clues on the relationships between nutritional problems and relevant factors; thus, further studies are needed to verify these relationships. However, through rigorous surveys and scientific sampling methods, a large regional representative sample of participants among eastern Chinese children and adolescents can present findings on multiple nutritional issues.

## 5. Conclusions

The presence of the double burdens of malnutrition are common among school-age children and adolescents in Jiangsu Province in eastern China. Demographic characteristics, behavior characteristics, parents’ characteristics, and family characteristics were associated with multiple malnutrition disorders. Health education, focusing on behavioral intervention and nutritional education of children, and on their living environments, may be a practical strategy in preventing multiple malnutrition disorders in school-age children and adolescents.

## Figures and Tables

**Table 1:** Basic characteristics of the research population.

Variables	Males (%)	Females (%)	All *n* = 3025	t or χ^2^ Values	*p* Value
	*n*= 1520	*n* = 1505			
Age (years)	11.4 ± 3.0	11.4 ± 3.0	11.4 ± 3.0	0.074	0.941
Age group				0.089	0.765
7–12 years	990 (65.1)	988 (65.6)	1978 (65.4)		
13–17 years	530 (34.9)	517 (34.4)	1047 (34.6)		
Living area				0.001	0.982
Urban	1272 (83.7)	1259 (83.7)	2531 (83.7)		
Rural	248 (16.3)	246 (16.3)	494 (16.3)		
Physical activity				0.613	0.434
Low	907 (59.7)	919 (61.1)	1826 (60.4)		
High	613 (40.3)	586 (38.9)	1199 (39.6)		
Screen time				3.432	0.064
Low	1320 (86.8)	1340 (89.0)	2660 (87.9)		
High	200 (13.2)	165 (11.0)	365 (12.1)		
Age of mother				0.170	0.680
≤37 (median)	783 (51.5)	764 (50.8)	1547 (51.1)		
≥38	737 (48.5)	741 (49.2)	1478 (48.9)		
Age of father				0.389	0.533
≤39 (median)	806 (53.0)	781 (51.9)	1587 (52.5)		
≥40	714 (47.0)	724 (48.1)	1438 (47.5)		
Education of mother				1.900	0.387
Primary school and below	226 (14.9)	206 (13.7)	432 (14.3)		
Middle school	1007 (66.3)	989 (65.7)	1996 (66.0)		
University and above	287 (18.9)	310 (20.6)	597 (19.7)		
Education of father				0.908	0.635
Primary school and below	134 (8.8)	119 (7.9)	253 (8.4)		
Middle school	1027 (67.6)	1034 (68.7)	2061 (68.1)		
University and above	359 (23.6)	352 (23.4)	711 (23.5)		
Household size				6.907	0.009
≤4 members	924 (60.8)	844 (56.1)	1768 (58.4)		
≥5 members	596 (39.2)	661 (43.9)	1257 (41.6)		

**Table 2:** Nutritional and biochemistry indicators for the research population.

Variables	Males Median (IQR)	Females Median (IQR)	All Median (IQR)	Z Values	*p* Value
Anthropometric measurements					
Height (cm)	152.1 (138.4, 168.0)	152.0 (138.0, 160.3)	152.1 (138.1, 162.6)	−5.826	<0.001
Weight (kg)	45.0 (32.6, 59.4)	42.2 (30.9, 52.1)	43.5 (31.7, 55.3)	−6.791	<0.001
WC (cm)	66.4 (59.1, 75.1)	62.5 (56.1, 68.5)	64.0 (57.7, 71.5)	−11.076	<0.001
SBP (mmHg)	116.8 (108.3, 125.5)	113.0 (105.3, 120.5)	115.0 (106.7, 122.7)	−9.233	<0.001
DBP (mmHg)	67.3 (62.0, 73.3)	67.7 (62.7, 73.3)	67.7 (62.3, 73.3)	−1.155	0.248
Biochemical indexes					
FBG (mmol/L)	5.29 (5.00, 5.59)	5.16 (4.90, 5.45)	5.22 (4.94, 5.52)	−7.172	<0.001
TG (mmol/L)	0.77 (0.60, 1.06)	0.83 (0.65, 1.09)	0.80 (0.62, 1.06)	−5.747	<0.001
TC (mmol/L)	3.95 (3.50, 4.49)	4.03 (3.63, 4.54)	4.00 (3.59, 4.52)	−3.282	0.001
LDL (mmol/L)	2.16 (1.80, 2.55)	2.21 (1.88, 2.59)	2.19 (1.84, 2.57)	−2.448	0.014
HDL (mmol/L)	1.56 (1.30, 1.84)	1.60 (1.37, 1.87)	1.59 (1.34, 1.86)	−2.801	0.005
Serum uric acid (μmol/L)	336.0 (276.0, 405.0)	297.0 (254.0, 339.0)	310.0 (260.0, 369.0)	−13.106	<0.001
Vitamin A (μg/mL)	0.38 (0.32, 0.43)	0.38 (0.32, 0.43)	0.38 (0.32, 0.43)	−0.046	0.963
Vitamin D (ng/mL)	16.1 (12.8, 20.1)	14.5 (11.4, 18.1)	15.3 (12.0, 19.1)	−8.368	<0.001
Zinc (μg/dL)	94.0 (87.0, 102.0)	93.0 (85.0, 99.0)	93.0 (86.0, 101.0)	−4.386	<0.001

IQR = interquartile range; WC = waist circumference; SBP = systolic blood pressure; DBP =: diastolic blood pressure; FBG = fasting blood glucose; TG = triglyceride; TC = total cholesterol; LDL = low-density lipoprotein; HDL = high-density lipoprotein.

**Table 3:** Prevalence of various categories of nutritional status among school-age children and adolescents in Jiangsu.

Nutritional Status	Males *n* (%)	Female n (%)	Total *n* (%)	χ^2^ Values	*p* Value
Weight groups				102.298	<0.001
Wasting	96 (6.3)	70 (4.7)	166 (5.5)		
Normal weight	893 (58.8)	1136 (45.5)	2029 (67.1)		
Overweight	271 (17.8)	176 (11.7)	447 (14.8)		
Obesity	260 (17.1)	123 (8.2)	383 (12.7)		
MetS				6.215	0.013
No	1427 (93.9)	1443 (95.9)	2870 (94.9)		
Yes	93 (6.1)	62 (4.1)	155 (5.1)		
Abdominal obesity				18.075	<0.001
No	1190 (78.3)	1269 (84.3)	2459 (81.3)		
Yes	330 (21.7)	236 (15.7)	566 (18.7)		
High TG				1.403	0.236
No	1304 (85.8)	1268 (84.3)	2572 (85.0)		
Yes	216 (14.2)	237 (15.7)	453 (15.0)		
Low HDL				3.160	0.075
No	95.3 (1448)	1453 (96.5)	2901 (95.9)		
Yes	72 (4.7)	52 (3.5)	124 (4.1)		
Elevated BP				0.159	0.690
No	887 (58.4)	889 (59.1)	1179 (58.7)		
Yes	633 (41.6)	616 (40.9)	1249 (41.3)		
Elevated FBG				15.868	<0.001
No	1446 (95.1)	1472 (97.8)	2918 (96.5)		
Yes	74 (4.9)	33 (2.2)	107 (3.5)		
Hyperuricemia				194.297	<0.001
No	897 (59.0)	1236 (82.1)	2133 (70.5)		
Yes	623 (41.0)	269 (17.9)	892 (29.5)		
High LDL				0.432	0.511
No	1451 (95.5)	1429 (95.0)	2880 (95.2)		
Yes	69 (4.5)	76 (5.0)	145 (4.8)		
High TC				7.727	0.005
No	1427 (93.9)	1373 (91.2)	2800 (92.6)		
Yes	93 (6.1)	132 (8.8)	225 (7.4)		
Vitamin A				0.049	0.976
Sufficiency	1285 (84.5)	1272 (84.5)	2557 (84.5)		
Marginal deficiency	222 (14.6)	219 (14.6)	441 (14.6)		
Deficiency	13 (0.9)	14 (0.9)	27 (0.9)		
Vitamin D				68.349	<0.001
Sufficiency	383 (25.2)	233 (15.5)	616 (20.4)		
Inadequacy	835 (54.9)	815 (54.2)	1650 (54.5)		
Deficiency	302 (19.9)	457 (30.4)	759 (25.1)		
Anemia				31.863	<0.001
No	1490 (98.0)	1415 (94.0)	2905 (96.0)		
Yes	30 (2.0)	90 (6.0)	120 (4.0)		
Zinc deficiency				3.417	0.065
No	1458 (95.9)	1422 (94.5)	2880 (95.2)		
Yes	62 (4.1)	83 (5.5)	145 (4.8)		

MetS = metabolic syndrome; high TG = high triglyceride; low HDL = low high-density lipoprotein; elevated BP = elevated blood pressure; elevated FBG = elevated fasting blood glucose; high LDL = high low-density lipoprotein; high TC = high total cholesterol.

**Table 4:** Multivariate logistic regression analysis of various nutritional disorders among school-age children and adolescents in Jiangsu Province.

	Metabolic Syndrome		Hyperuricemia		Vitamin A Insufficiency		Vitamin D Deficiency		Anemia		Zinc Deficiency	
Characteristics	AOR (95%CI)	*p* Value	AOR (95%CI)	*p* Value	AOR (95%CI)	*p* Value	AOR (95%CI)	*p* Value	AOR (95%CI)	*p* Value	AOR (95%CI)	*p* Value
Sex												
Males (reference)	1.00	--	1.00	--	1.00	--	1.00	--	1.00	--	1.00	--
Females	1.20 (0.83–1.74)	0.331	0.30 (0.25–0.36)	<0.001	0.91 (0.74–1.12)	0.378	1.80 (1.51–2.15)	<0.001	2.95 (1.93–4.51)	<0.001	1.33 (0.94–1.88)	0.102
Age group												
7–12 years (reference)	1.00	--	1.00	--	1.00	--	1.00	--	1.00	--	1.00	--
13–17 years	1.79 (1.17–2.73)	0.007	5.09 (4.13–6.27)	<0.001	0.34 (0.26–0.45)	<0.001	2.53 (2.09–3.08)	<0.001	1.82 (1.20–2.77)	0.005	0.94 (0.63–1.41)	0.774
Living area												
Urban	1.00	--	1.00	--	1.00	--	1.00	--	1.00	--	1.00	--
Rural	0.76 (0.43–1.34)	0.345	0.97 (0.75–1.25)	0.796	1.24 (0.95–1.62)	0.116	1.14 (0.89–1.44)	0.298	1.43 (0.89–2.31)	0.142	1.80 (1.21–2.68)	0.004
Weight group												
Others (reference)	1.00	--	1.00	--	1.00	--	1.00	--	1.00	--	1.00	--
Overweight	7.47 (4.26–13.11)	<0.001	1.94 (1.53–2.46)	<0.001	0.63 (0.46–0.87)	<0.001	1.02 (0.80–1.31)	0.877	0.71 (0.39–1.28)	0.255	0.78 (0.46–1.32)	0.357
Obesity	41.14 (25.05–67.56)	<0.001	4.38 (3.38–5.67)	<0.001	0.37 (0.25–0.55)	0.005	0.85 (0.64–1.13)	0.269	0.37 (0.15–0.93)	0.034	0.81 (0.45–1.44)	0.466
Physical activity												
Low	1.00	--	1.00	--	1.00	--	1.00	--	1.00	--	1.00	--
High	1.31 (0.91–1.88)	0.146	1.19 (0.99–1.43)	0.061	0.93 (0.75–1.16)	0.528	0.69 (0.58–0.83)	<0.001	1.07 (0.73–1.56)	0.746	1.25 (0.89–1.77)	0.204
Screen time												
Low	1.00	--	1.00	--	1.00	--	1.00	--	1.00	--	1.00	--
High	0.60 (0.33–1.10)	0.097	0.91 (0.69–1.19)	0.487	1.45 (1.09–1.94)	0.011	1.40 (1.09–1.80)	0.008	1.25 (0.73–2.13)	0.420	0.78 (0.46–1.32)	0.360
Age of mother												
≤36	1.00	--	1.00	--	1.00	--	1.00	--	1.00	--	1.00	--
≥37	0.93 (0.55–1.55)	0.775	1.46 (1.13–1.89)	0.004	0.78 (0.58–1.06)	0.113	1.13 (0.88–1.45)	0.349	1.34 (0.78–2.29)	0.290	0.88 (0.53–1.45)	0.621
Age of father												
≤36	1.00	--	1.00	--	1.00	--	1.00	--	1.00	--	1.00	--
≥37	0.97 (0.58–1.63)	0.901	0.94 (0.73–1.23)	0.668	1.07 (0.79–1.45)	0.662	1.03 (0.80–1.33)	0.788	0.75 (0.44–1.27)	0.285	0.82 (0.49–1.36)	0.443
Education of mother												
Primary school and below	1.00	--	1.00	--	1.00	--	1.00	--	1.00	--	1.00	--
Middle school	1.08 (0.58–2.03)	0.804	0.94 (0.71–1.25)	0.693	0.64 (0.47–0.88)	0.005	0.68 (0.52–0.89)	0.004	1.27 (0.70–2.29)	0.438	0.95 (0.58–1.56)	0.847
University and above	1.09 (0.47–2.50)	0.847	1.23 (0.82–1.83)	0.315	0.55 (0.35–0.89)	0.014	0.58 (0.40–0.86)	0.006	1.03 (0.43–2.45)	0.947	0.48 (0.20–1.16)	0.104
Education of father												
Primary school and below	1.00	--	1.00	--	1.00	--	1.00	--	1.00	--	1.00	--
Middle school	0.92 (0.45–1.87)	0.817	0.86 (0.61–1.22)	0.397	1.10 (0.74–1.64)	0.626	1.17 (0.84–1.62)	0.364	1.06 (0.51–2.20)	0.886	0.72 (0.41–1.28)	0.268
University and above	0.58 (0.24–1.40)	0.228	0.63 (0.41–0.97)	0.035	0.73 (0.43–1.22)	0.223	0.97 (0.63–1.48)	0.883	1.03 (0.51–2.20)	0.942	0.47 (0.20–1.09)	0.078
Household size												
≤4 members	1.00	--	1.00	--	1.00	--	1.00	--	1.00	--	1.00	--
≥5 members	1.20 (0.83–1.73)	0.327	0.93 (0.77–1.12)	0.465	1.20 (0.97–1.47)	0.088	1.25 (1.05–1.49)	0.014	1.10 (0.75–1.62)	0.609	1.30 (0.92–1.83)	0.136

**Table 5:** Multivariate Poisson regression analysis of overnutrition- and undernutrition-related disorders among school-age children and adolescents in Jiangsu Province.

	Overnutrition-Related Disorders		Undernutrition-Related Disorders	
Characteristics	IRR (95%CI)	*p* Value	IRR (95%CI)	*p* Value
Sex				
Males (reference)	1.00	--	1.00	--
Females	0.81 (0.51–0.85)	<0.001	1.33 (1.20–1.48)	<0.001
Age group				
7–12 years (reference)	1.00	--	1.00	--
13–17 years	1.44 (1.35–1.54)	<0.001	1.17 (1.04–1.32)	0.008
Living area				
Urban	1.00	--	1.00	--
Rural	1.00 (0.91–1.08)	0.939	1.21 (1.06–1.38)	0.005
Weight group				
Others (reference)	1.00	--	1.00	--
Overweight	1.66 (1.53–1.79)	<0.001	0.86 (0.73–1.00)	0.05
Obesity	2.77 (2.58–2.97)	<0.001	0.67 (0.55–0.81)	<0.001
Physical activity				
Low	1.00	--	1.00	--
High	1.05 (0.99–1.12)	0.095	0.89 (0.80–0.99)	0.027
Screen time				
Low	1.00	--	1.00	--
High	0.94 (0.86–1.03)	0.202	1.23 (1.06–1.42)	0.005
Age of mother				
≤37	1.00	--	1.00	--
≥38	1.07 (0.98–1.16)	0.122	0.99 (0.86–1.15)	0.946
Age of father				
≤36	1.00	--	1.00	--
≥37	0.98 (0.90–1.07)	0.658	0.99 (0.85–1.15)	0.852
Education of mother				
Primary school and below	1.00	--	1.00	--
Middle school	0.99 (0.90–1.08)	0.759	0.81 (0.70–0.94)	0.006
University and above	1.03 (0.91–1.18)	0.631	0.68 (0.54–0.89)	0.001
Education of father				
Primary school and below	1.00	--	1.00	--
Middle school	1.01 (0.90–1.13)	0.913	1.04 (0.86–1.26)	0.689
University and above	0.90 (0.77–1.04)	0.135	0.83 (0.65–1.07)	0.147
Household size				
≤4 members	1.00	--	1.00	--
≥5 members	1.02 (0.96–1.09)	0.435	1.17 (1.05–1.29)	0.004

Overnutrition-related disorders: the prevalent five MetS components and hyperuricemia, high LDL, and high TC. Undernutrition-related disorders: prevalent vitamin A insufficiency, vitamin D deficiency, anemia, and zinc deficiency. IRR: incidence rate rations. 95%CI: 95% confidence interval.

## Data Availability

The datasets used and/or analyzed during this study are available from the corresponding author on reasonable request.

## Supplementary Materials

The following supporting information can be downloaded at https://www.mdpi.com/article/10.3390/nu14040758/s1: Table S1: Prevalence of various nutritional status in Jiangsu Province children and adolescents aged 7–12 years and 13–17 years.
