# Supplementary material for: Multilevel Analysis of the Nutritional and Health Status among Children and Adolescents in Eastern China
Source: Nutrients. 2022 Feb 11;14(4):758. doi: 10.3390/nu14040758 (PMC8877382; doi:10.3390/nu14040758)
Supplement: Supplementary file 1 [file nutrients-14-00758-s001.zip › nutrients-1574142-supplementary.pdf]

Supplementary Materials:

Table S1. Prevalence of various nutritional status in Jiangsu Province children and adolescents aged 7-12 years and 13-17 years.

Nutritional status	7-12 years group					13-17 years group					χ^2 values (between age groups)	*p* values (between age groups)
	Males N (%)	Female N (%)	Total N (%)	χ^2 values	*p* value	Males N (%)	Female N (%)	Total N (%)	χ^2 values	*p* values		
Weight groups				66.997	＜0.001				56.647	＜0.001	17.904	＜0.001
Wasting	61(6.2)	60(6.1)	121(6.1)			35(6.6)	10(1.9)	45(4.3)				
Normal weight	569(57.5)	730(73.9)	1299(65.7)			324(61.1)	406(78.5)	730(69.7)				
Over weight	179(18.1)	99(10.0)	278(14.1)			92(17.4)	77(14.9)	169(16.1)				
Obesity	181(18.3)	99(10.0)	280(14.2)			79(14.9)	24(4.6)	103(9.8)				
Metabolic syndrome				0.014	0.907				16.724	＜0.001	2.096	0.148
No	944(95.4)	941(95.2)	1885(95.3)			483(91.1)	502(97.1)	985(94.1)				
Yes	46(4.6)	47(4.8)	93(4.7)			47(8.9)	15(2.9)	62(5.9)				
Abdominal obesity				5.675	0.017				17.242	＜0.001	9.170	0.002
No	768(77.6)	809(81.9)	1577(79.7)			422(79.6)	460(89.0)	882(84.2)				
Yes	222(22.4)	179(18.1)	401(20.3)			108(20.4)	57(11.0)	165(15.8)				
High TG				2.090	0.148				0.010	0.921	4.685	0.030
No	863(87.2)	839(84.9)	1702(86.0)			441(83.2)	429(83.0)	870(83.1)				
Yes	127(12.8)	149(15.1)	276(14.0)			89(16.8)	88(17.0)	177(16.9)				
Low HDL				0.064	0.801				8.884	0.003	4.563	0.033
No	956(96.6)	952(96.4)	1908(96.4)			492(92.8)	501(96.9)	993(94.8)				
Yes	34(3.4)	36(3.6)	70(3.5)			34(3.4)	16(3.4)	54(5.2)				
Elevated blood pressure				0.162	0.688				1.737	0.188	22.641	＜0.001
No	555(56.1)	545(55.2)	1100(56.5)			332(62.6)	344(66.5)	676(64.6)				
Yes	435(43.9)	443(44.8)	878(44.4)			198(37.4)	173(33.5)	371(35.4)				
Elevated FBG				8.722	0.003				7.266	0.007	0.165	0.684
No	944(95.4)	966(97.8)	1910(96.6)			502(94.7)	506(97.9)	1008(96.3)				
Yes	46(4.6)	22(2.2)	68(3.4)			28(5.3)	11(2.1)	39(3.7)				
Hyperuricemia				29.517	＜0.001				245.458	＜0.001	337.742	＜0.001
No	761(76.9)	853(86.3)	1614(81.6)			136(25.7)	383(74.1)	519(49.6)				
Yes	229(23.1)	135(13.7)	364(18.4)			394(74.3)	134(25.9)	528(50.4)				
High LDL				0.508	0.476				0.006	0.936	3.321	0.068
No	941(95.1)	932(94.3)	1873(94.7)			510(96.2)	497(96.1)	1007(96.2)				
Yes	49(4.9)	56(5.7)	105(5.3)			20(3.8)	20(3.9)	40(3.8)				
High TC				1.817	0.178				8.832	0.003	1.671	0.196

No	920(92.9)	902(91.3)	1822(92.1)			507(95.7)	471(91.1)	978(93.4)				
Yes	70(7.1)	86(8.7)	156(7.9)			23(4.3)	46(8.9)	69(6.6)				
Vitamin A				2.231	0.328				8.052	0.018	73.328	＜0.001
Sufficiency	784(79.2)	807(81.7)	1591(80.4)			501(94.5)	465(89.9)	966(92.3)				
Inadequacy	195(19.7)	169(17.1)	364(18.4)			27(5.1)	50(9.7)	77(7.4)				
Deficiency	11(1.1)	12(1.2)	23(1.2)			2(0.4)	2(0.4)	4(0.4)				
Vitamin D				40.895	＜0.001				36.696	＜0.001	179.663	＜0.001
Sufficiency	304(30.7)	205(20.7)	509(25.7)			79(14.9)	28(5.4)	107(10.2)				
Inadequacy	547(55.3)	555(56.2)	1102(55.7)			288(54.3)	260(50.3)	548(52.3)				
Deficiency	139(14.0)	228(23.1)	367(18.6)			163(30.8)	229(44.3)	392(37.4)				
Anemia				5.377	0.020				33.232	＜0.001	10.396	0.001
No	964(97.8)	947(95.9)	1911(96.9)			521(98.5)	467(90.3)	988(84.5)				
Yes	22(2.2)	40(4.1)	62(3.1)			8(1.5)	50(9.7)	58(5.5)				
Zinc deficiency				0.536	0.464				4.828	0.028	0.561	0.454
No	944(95.4)	935(94.6)	1879(95.0)			514(97.0)	487(94.2)	1001(95.6)				
Yes	46(4.6)	53(5.4)	99(5.0)			16(3.0)	30(5.8)	46(4.4)				

high TG = high triglyceride; low HDL = low high-density lipoprotein; elevated FBG = elevated fasting blood glucose; high LDL = high low-density lipoprotein; high TC = high total cholesterol.
